# Dietary Habits and Intestinal Immunity: From Food Intake to CD4^+^ T_*H*_ Cells

**DOI:** 10.3389/fimmu.2018.03177

**Published:** 2019-01-15

**Authors:** Francesco Siracusa, Nicola Schaltenberg, Eduardo J. Villablanca, Samuel Huber, Nicola Gagliani

**Affiliations:** ^1^Department of General, Visceral and Thoracic Surgery, University Medical Center Hamburg-Eppendorf, Hamburg, Germany; ^2^Department of Biochemistry and Molecular Cell Biology, University Medical Center Hamburg-Eppendorf, Hamburg, Germany; ^3^Immunology and Allergy Unit, Department of Medicine Solna, Karolinska Institute and University Hospital, Stockholm, Sweden; ^4^Department of Medicine, University Medical Center Hamburg-Eppendorf, Hamburg, Germany

**Keywords:** inflammation, mucosal immunity, CD4 T cells, western diet, fat, salt, fiber, microbiota

## Abstract

Dietary habits have a profound impact on intestinal homeostasis and in general on human health. In Western countries, high intake of calories derived from fried products, butter and processed meat is favored over dietary regimens rich in fruits and vegetables. This type of diet is usually referred to as Western-type diet (WTD) and it has been associated with several metabolic and chronic inflammatory conditions of the gastrointestinal tract. In this review, we describe how WTD promotes intestinal and extra-intestinal inflammation and alters mucosal immunity acting on CD4^+^ T cells in a microbiota-dependent or –independent fashion, ultimately leading to higher susceptibility to infectious and autoimmune diseases. Moreover, summarizing recent findings, we propose how dietary supplementation with fiber and vitamins could be used as a tool to modulate CD4^+^ T cell phenotype and function, ameliorating inflammation and restoring mucosal homeostasis.

## Introduction

In the gastrointestinal (GI) tract, the immune system is constantly under great environmental pressure, continuously facing a wide variety of antigens derived both from intestinal microbiota and from food. Intestinal CD4^+^ T helper (T_H_) cells are key mediators of mucosal immunity, and according to their effector functions, they can be divided into different populations, namely T_H_1, T_H_2, and T_H_17, with the T_H_17 cells being relatively abundant within the GI tract (1–4). Here, different bacterial species dictate whether intestinal CD4^+^ T cells acquire pro- or anti-inflammatory effector phenotypes (5, 6), highlighting the crucial function of the intestinal microbiota in maintaining mucosal homeostasis. Pro-inflammatory responses driven by T_H_ cells are controlled by different subsets of CD4^+^ T cells with regulatory capacities, namely T_reg_ and T_R_1 cells, key players in promoting and maintaining mucosal tolerance to self- and food-related antigens (7–9). However, when mucosal tolerance fails to limit pro-inflammatory immune responses, this results in intestinal inflammation which can lead to the development of immune-mediated inflammatory diseases (IMIDs) such as inflammatory bowel diseases (IBDs). IBDs are among the leading diseases in Western countries (10, 11) and the observation that T_H_17 cells and T_H_17-associated cytokines such as IL-17A, IL-17F, and IL-22 are generally enriched in the inflamed mucosa of IBD patients, suggests that T_H_17 cells drive intestinal inflammation (12, 13). Interestingly, CD4^+^ T cells, especially T_H_17 cells, are highly susceptible to components of Western-type diet (WTD) (14, 15), and WTD has been associated with higher incidence of IBDs (16). Moreover, high intake of calories derived from processed meat, butter and fried products, all components of WTD, have been described to instantly alter the composition of the intestinal microbiota, a phenomenon called dysbiosis, toward a lower *Bacteroidetes* to *Firmicutes* ratio (17–19). Dysbiosis is also commonly found among patients suffering from IMIDs, including IBDs (20–23). However, despite these strong associations, a direct cause/effect link between WTD and development of IBDs has not yet been proven.

**Figure 1:**
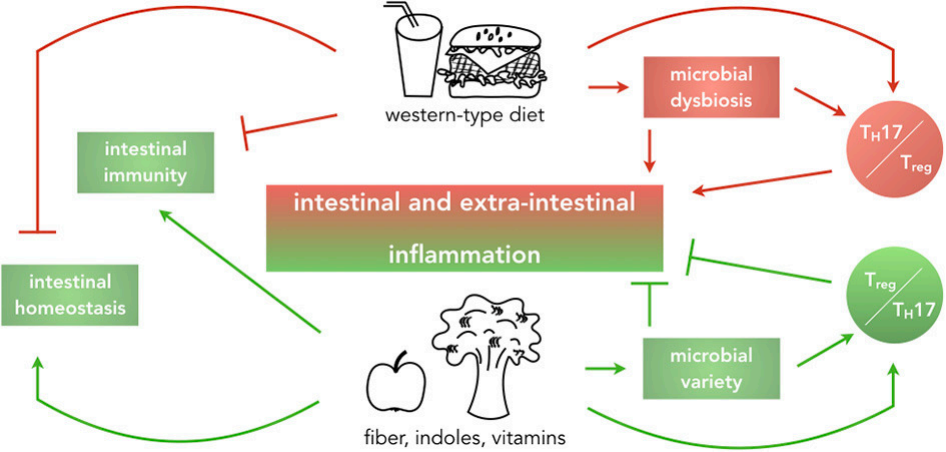
Impact of dietary habits on local and systemic homeostasis and immunity. Graphical abstract summarizing the main findings that this review will discuss. Western-type diets, rich in fat, cholesterol, sugar, and salt are reported to drive intestinal and extra-intestinal inflammation by causing microbial dysbiosis and alteration of the balance of pro- and anti-inflammatory T cells in the intestine, ultimately dampening intestinal immunity and affecting intestinal homeostasis. In contrast, diets enriched in fiber, indoles and vitamins implement beneficial effects on intestinal homeostasis by increasing microbial variety and inducing a regulatory environment.

In this review, we discuss how changes in dietary habits favoring WTD affect intestinal immunity by altering composition of the intestinal microbiota and phenotype and functions of effector and regulatory CD4^+^ T cells. Furthermore, we propose that WTD leads both to higher susceptibility to infections and higher incidence of chronic autoimmune diseases, thus exacerbating intestinal and extra-intestinal inflammation. We support the hypothesis that supplementation of diets with defined products of bacterial or dietary origin can ameliorate WTD-induced inflammation, acting on the effector/regulatory T cell axis and, in turn, restoring intestinal homeostasis (Figure 1). The findings presented in this review are mostly based on murine experiments and are cross-validated in humans, where possible.

## The Intestinal Immune System and the Microbiota

The intestinal immune system promotes mucosal immunity and maintains tolerance to dietary and microbial antigens, both through its innate and adaptive components located within intestinal epithelia and lamina propria.

In addition to M cells and intraepithelial lymphocytes (IELs), goblet cells, Paneth cells and innate lymphoid type 3 cells (ILC3s) constitute the innate arm of the intestinal immune system. On the other hand, antibody-secreting plasma cells, CD4^+^ and CD8^+^ T cells represent the intestinal adaptive immune system. Mucins secreted by goblet cells form the single mucus layer of the small intestine and the two-layered mucus of the colon with the inner layer being impermeable to bacteria (24). ILC3s efficiently contribute to intestinal homeostasis through secretion of IL-17 and IL-22 (25, 26) that instruct Paneth cells to secrete antimicrobial peptides (AMPs) into the intestinal lumen. Although the innate components of the intestinal immune system are fundamental in providing a first line of protection from invading microbes, this review focuses on CD4^+^ T_H_ cells given their unique role in orchestrating adaptive immune responses, protecting from infections.

Among the different CD4 T_H_ cell subsets, T_H_17 cells are relatively abundant within the GI tract (27). They are characterized by the expression of the master transcription factor RORγt, the chemokine receptor CCR6 and the transcription factor aryl hydrocarbon receptor (AhR) (28, 29). T_H_17 cells secrete the highest amount of IL-17 and IL-22, contributing to protection against fungal and bacterial infections, ultimately maintaining mucosal immunity (1). However, the observation that high levels of IL-17 and IL-22 are found in the inflamed mucosa of patients suffering from IBDs, highlights their dualistic role in limiting or promoting intestinal inflammation (12, 13). Complete blockage of IL-17A failed to ameliorate intestinal inflammation in Crohn's disease, which might be explained by preventing the beneficial actions of IL-17A, such as promotion of AMP production that ultimately protects the host against invading microbes (30). In line with this, it has been also shown that IL17-secreting TCRγδ^+^ T cells mediate gut permeability and exert a protective function on epithelial barrier integrity (31). At the same time IL-17A-deficient T cells have been shown to induce a more aggressive disease outcome in a mouse model of transfer colitis (32). Taken together, these findings suggest that the cellular source of IL-17A production might determine the beneficial or detrimental role of the cytokine itself. Therefore, cell-specific targeting of IL-17A could open new therapeutic approaches. Furthermore, it has been described that T_H_17 cells are a highly plastic cell population, able to acquire properties typical of other CD4^+^ T cell subsets (33). Due to their high plasticity, T_H_17 cells can be either beneficial or detrimental to the host according to the cytokine profile they exhibit in response to inflammatory stimuli. While IL-12 and IL-23 drive the conversion of T_H_17 cells into pro-inflammatory T_H_1 cells, inducing acquisition of T-bet and CXCR3 and secretion of IFN-γ (33, 34), exposure to TGF-β and AhR ligands mediate the acquisition of IL-10 secretion from T_H_17, thus converting them into anti-inflammatory T_R_1^exTH17^ cells (35, 36). Characterized by lack of Foxp3 and expression of the co-inhibitory receptors CD49b and LAG-3 (37), both *bona fide* and T_H_17-derived T_R_1 cells (i.e., T_R_1^exTH17^) limit expansion of pro-inflammatory T_H_17 cells in an IL-10-dependent manner (38). In addition, pathogenicity of T_H_17 cells is also controlled by Foxp3^+^ T_reg_ cells (38), which, similarly to T_R_1 and T_H_17 cells, are relatively abundant in the small intestine, where most of the dietary products are absorbed (9). In summary, pro- and anti-inflammatory CD4^+^ T cells co-exist within the GI tract being subject to a highly dynamic microenvironment.

An additional layer of complexity to this tight balance of pro- and anti-inflammatory cells is added by the microbiota, whose composition and abundance vary along the GI tract (39–41). It is increasingly recognized that the intestinal microbiota exerts non-redundant functions in the maintenance of homeostasis of the host, ranging from synthesis of nutrients to protection against invading pathogens and modulation of immune responses (42–44). Indeed, studies on germ-free (GF) mice have underlined a higher susceptibility to viral or bacterial infections of mice deprived of their intestinal microbiota as compared to mice housed under specific pathogen free (SPF) conditions (45, 46). This is probably due to the fact that bacterial species dictate the phenotype of CD4^+^ T cells. For example, *Bacteroides fragilis* favor differentiation of naïve CD4^+^ T cells toward IFN-γ-producing T_H_1 cells (6), while *Segmented Filamentous Bacteria* (SFB) drives differentiation toward IL-17-secreting T_H_17 cells (5). Of note, presence of SFB within the intestinal microbiota prevents the growth of pathogenic *Citrobacter rodentium*, probably due to T_H_17 induction, ameliorating colonic inflammation (5). These findings highlight once more the dualistic nature of T_H_17 cells in preventing or exacerbating intestinal inflammation.

Composition of the intestinal microbiota and in turn, intestinal CD4^+^ T cells are therefore key players in promoting mucosal homeostasis. On the one hand, bacterial species are able to shape intestinal immune functions by modulating CD4^+^ T cell responses. On the other hand, while T_H_17 cells mediate immunity to invading microbes, Foxp3^+^ T_reg_ and T_R_1 cells maintain tolerance to self and dietary antigens, preventing, as well, uncontrolled T_H_17 cell-mediated immune responses. Failure to suppress uncontrolled CD4^+^ T_H_ cell-mediated immune responses may lead to IBDs, as seen in mice lacking critical immunosuppression-associated genes, such as IL-10, which develop spontaneous colitis (47). In agreement, IBDs have been defined by aberrant CD4^+^ T_H_ cell responses against the commensal microbiota in genetically susceptible hosts (48). How commensal-specific CD4^+^ T_H_ cell responses develop has been reviewed elsewhere (49).

## Dietary Habits in Western Countries

Dietary habits have a profound impact on the lifestyle of individuals. High lipid content in WTD often derived from saturated fatty acids and cholesterol, in addition to excess intake of sugar is linked to higher incidence of colorectal cancer and IMIDs (50–52). Additionally, elevated salt intake and consumption of medium (MCFA) and long chain fatty acids (LCFA), such as lauric and palmitic acids, induce or exacerbate inflammation, acting on the intestinal microbiota, as well as on the innate and adaptive components of the intestinal immune system (53–55).

WTD-favoring dietary habits are also in line with a reduced absorption of vitamins and intake of vegetables and fruits rich in fiber. Dietary fiber consists of non-starch polysaccharides, cellulose, lignin and other plant-derived oligo- or polysaccharides that are not digestible or absorbable in the small intestine (56). It is accepted that diets rich in fiber are beneficial to the host, and dietary regimens favoring consumption of fiber have been associated with a decreased risk of type 2 diabetes (T2D), cardiovascular diseases and intestinal inflammation (57–59). This suggests that fiber can potentially modulate intestinal related and unrelated immune responses. However, how the fiber ameliorates inflammation remains poorly understood. One possible mechanism could reside in its fermentation by bacteria within the colon, which results in the production of short-chain fatty acids (SCFAs) (60, 61). Indeed, acetate, butyrate and propionate, all SCFAs, mediate beneficial effects on the host by engagement of G protein–coupled receptors (GPRs) expressed by a variety of cells, including intestinal CD4^+^ T cells (62). In addition, recent evidences suggest that the beneficial effects of fiber consumption on the host might reside in the changes it induces in the composition of the intestinal microbiota itself (63).

In short, dietary habits greatly influence human health, modulating function of CD4^+^ T cells and composition of intestinal microbiota.

## WTD Induces Mucosal Inflammation Altering Immunity

### Lipids, Cholesterol, and Salt

In this part of the review, we describe the effects that high intake of lipids, cholesterol and salt have on the intestinal immune system, dissecting the complex interplay between adaptive immune cells and the intestinal microbiota. We then summarize recent findings on how WTD-favoring dietary regimens increase susceptibility to chronic autoimmune diseases and infections with commensal bacteria (Table 1). Ultimately, we propose how intestinal and extra-intestinal inflammation driven by WTD can be modulated by supplementation of defined bio-products of microbial or dietary origin, which in turn act on CD4^+^ T cells.

**Table 1:** Table showing how different components of WTD drive cellular and functional phenotypes associated with intestinal and extra-intestinal inflammation.

**Components of WTD**	**Intestinal inflammation**	**Effects on CD4^**+**^ T cells**	**Effects on gut microbiota**	**Susceptibility to infection/diseases**
High fat	↑ colonic IL-1β, IL-6, TNF-α (64, 65) ↑ gut permeability (66) ↓ mucus layers (67, 68)	↑ Th1 cells (69)	↑ Proteobacteria ↑Firmicutes ↓Bacteroidetes (17–19, 70)	↑*Bilophila wadsworthia* (69) ↑ invasive *E. coli* (71, 72)
High salt	↑ colitis (54, 55)	↑ Th17 cells (15) ↓ inhibitory Treg cells (73)	↓*Lactobacillus* spp. (14, 54)	↑ colitis (54, 55) ↑ EAE (15) ↑ GVHD (73)
High cholesterol	↑ small intestine IL-1β, CD11b^+^ myeloid cells (74)	Not reported	↑*Bilophila wadsworthia* (69)	↑ ANA ↑ T cell priming ↑ B cell expansion (75)
High LCFA	Not reported	↑ Th1, Th17 cells (53)	↓*Prevotellaceae*	↑ EAE (53)
			↓ S24-7 families (53)	

*ANA, Anti-Nuclear Antibodies; EAE, Experimental Autoimmune Encephalomyelitis; GVHD, Graft-vs.-Host Disease; LCFA, Long-Chain Fatty Acid*.

**Figure 2:**
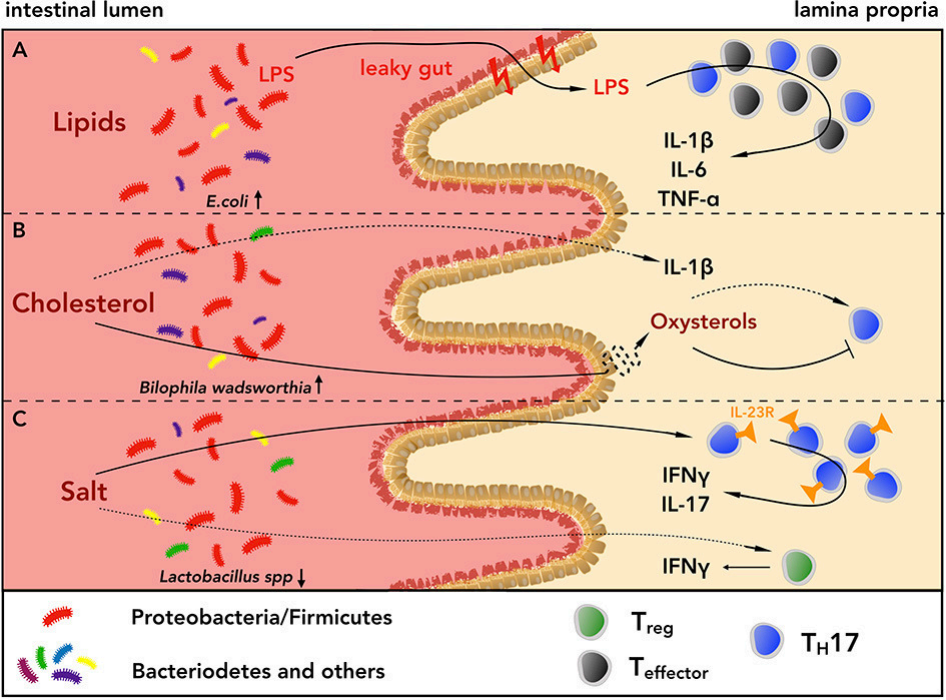
Lipids, cholesterol and salt shape the intestinal CD4^+^ T cell phenotype in a microbiota-dependent or independent-manner. **(A)** High intake of lipids induces dysbiosis, shifting the composition of the intestinal microbiota toward a higher ratio of *Proteobacteria* and *Firmicutes* to *Bacteroidetes*. This can lead to higher susceptibility to pathobiont infections, e.g. from invasive *E. coli* or *Bilophila wadsworthia* (BW). The mucus layers of both small intestine and colon get thinned, leading to higher gut permeability, which in turn favors the invasion of Gram-negative bacteria, and exacerbates intestinal inflammation. Furthermore, diets rich in saturated fatty acids increase the levels of pro-inflammatory cytokines, such as IL-1β, IL-6, TNF-α, within the gastrointestinal (GI) tract, contributing to the inflammatory state. **(B)** Diets rich in cholesterol alter the composition of bile acids and increase the levels of IL-1β in the small intestine, creating an inflammatory environment, which can lead to higher susceptibility to infections with BW, a pathogen known to require bile acids to outgrow. Cholesterol metabolites can also modulate intestinal inflammation inhibiting or promoting T_H_17 cell development through interaction with LXR or RORγt, respectively. **(C)** Within the WTD-driven intestinal inflammation, salt can alter the phenotype of CD4^+^ T_H_17 and T_reg_ cells either directly or indirectly, worsening mucosal homeostasis. Via triggering of serum glucocorticoid kinase-1 (SGK1), salt drives the expression of IL-23R on T_H_17 cells, inducing their pathogenicity, and it promotes IFN-γ secretion from T_reg_ cells, attenuating their suppressive capacities. Furthermore, high intake of salt can increase the frequencies of pathogenic T_H_17 cells reducing the amount of *Lactobacillus* spp. within the GI tract.

In addition to inducing systemic low-grade chronic inflammation typical of obesity (76, 77), WTD promotes local intestinal inflammation through a variety of mechanisms often linked to alteration of the intestinal microbiota composition (i.e., dysbiosis). Figure 2 provides a graphical summary.

High intake of fat increases the levels of IL-1β, IL-6, TNF-α, and NF-κB in the colon (64, 65), resulting in higher concentration of lipocalin 2 (Lcn2) in the feces, a biomarker of intestinal inflammation (78). Thinning of the mucus layers of the small intestine and colon (67, 68) and higher gut permeability (66) lead to increased presence of invading Gram-negative bacteria and higher plasma levels of LPS (79, 80), exacerbating local and systemic inflammation. These findings indicate that the high lipid content of WTD affects mucosal homeostasis, inducing thinning of the protective intestinal mucus layers and thus, increasing gut permeability and levels of pro-inflammatory cytokines.

Similarly, high intake of cholesterol increases levels of IL-1β in the small intestine of mice and frequencies of CD11b^+^ and CD11c^+^ cells (74). Along the same line, liver X receptor (LXR)-deficient mice (LXRαβ^−/−^), which lack the receptor for oxysterols (i.e. cholesterol metabolites), fed for 8 or 16 weeks with WTD showed higher titers of antinuclear antibodies (ANA), increased B cell numbers and augmented T_H_ cell priming, developing a lupus-like autoimmune disease (75). While these findings suggest that metabolism of cholesterol through LXR is crucial for preventing autoimmunity, the effect of excess intake of dietary cholesterol on T_H_ cells still remains unclear. Indeed, oxysterols have been shown to both favor and inhibit T_H_17 cell differentiation via direct binding to RORγt (81) or engagement of LXR (82) respectively, ascribing a context-dependent beneficial or detrimental role to cholesterol. High cholesterol levels might also increase the production of bile acids (BAs) and high BA concentration in the colon, together with BA malabsorption, has been suggested as a possible cause of diarrhea (83), a condition that is commonly present in patients suffering from IBDs. However, the etiology of diarrhea in IBD patients is still under debate and it can be ascribed to a sum of factors, including intestinal inflammation and disruption of barrier integrity, rather than to one single factor.

In addition to direct effects on CD4^+^ T cells, diets rich in lipids and cholesterol have been shown to drastically alter the composition of the intestinal microbiota. Of note, phenotype and effector functions of intestinal CD4^+^ T cells are strictly associated with the different bacterial species of the microbiota, and the intestinal microbiota quickly responds to changes in dietary regimens. Indeed, long-term feeding of mice with WTD shifts the composition of intestinal microbiota toward a higher ratio of *Proteobacteria* and *Firmicutes* species over *Bacteroidetes* (19, 70), leading to higher susceptibility to pathobiont infections by invasive *E. coli* (71, 72). Similarly, diets rich in saturated milk-derived fat favor the growth of the pathobiont *Bilophila wadsworthia* (BW) in the colon of IL-10^−/−^ mice, increasing the incidence of spontaneous colitis (69), and SPF mice colonized with BW present higher expression of IL-6 and Serum Amyloid A (SAA), exhibiting systemic inflammation (84). Of note, BW requires bile in its medium to be cultured (85), and diets rich in saturated milk-derived fat increased the amount of taurine conjugated bile acids (TCA) (69), indicating an as-of-yet undefined cross-talk between bile acid composition and intestinal microbiota.

These findings not only show that the intestinal microbiota is highly susceptible to perturbations of dietary regimes, but they also reveal a direct link between WTD, dysbiosis and increased susceptibility to infections and colitis. Further studies are needed to dissect how specific bacterial species act on the different CD4^+^ T cell populations.

Interestingly, dysbiosis is a common feature of patients suffering from IBDs, which is reflected by lower complexity of microbial species (86) and also exhibit increased frequencies of T_H_17 cells and amounts of IL-17 and IL-22 as compared to healthy individuals (12, 13).

It is however still unclear whether WTD first alters the composition of the intestinal microbiota that in turn induces pro-inflammatory T_H_17 cells and promotes intestinal inflammation, or vice versa. Moreover, the exact components of WTD able to target microbiota and/or T_H_17 cells still remain to be fully identified.

In the last years salt has been given a lot of attention*. Wu et al*. have shown that serum glucocorticoid kinase-1 (SGK1) drives the expression of IL-23R in T_H_17 cells via inactivation of Foxo1 (15), and it is known that IL-23R expression on T_H_17 cells defines their pathogenicity (87, 88). Interestingly, SGK1 has been shown to regulate salt sensing by different cell types, including epithelial colonic cells (89, 90). In their work, *Wu et al*. showed that mice fed with high salt diet (HSD) for 3 weeks exhibited higher frequencies of lamina propria (LP) T_H_17 cells as compared to normal chow-fed mice. Furthermore, HSD-fed mice were more susceptible to experimental autoimmune encephalomyelitis (EAE), showing prominent infiltration of T_H_17 in their central nervous system (CNS) (15). A similar phenotype has been described in mice fed with diets rich in lauric acid (53). Along the same line, HSD-fed mice presented increased intestinal inflammation in IL-10^−/−^ mice (55) and increased severity of Dextran Sulfate Sodium (DSS)- and 2,4-Dinitrobenzene Sulfonic Acid (DNBS)-induced colitis (54). These studies not only indicate that dietary habits favoring WTD act locally inducing intestinal inflammation, but they also suggest a link between dietary regimes and extra-intestinal inflammation. Furthermore, recent evidence in a pilot human study showed that 14 days high salt challenge increased the number of circulating IL-17A- and TNF-α-secreting T_H_17 cells. This was associated with higher blood pressure, which is a risk factor for atherosclerosis (14). High salt consumption has also been described to induce IFN-γ secretion from human T_reg_ cells, inhibiting their suppressive function both *in vitro* and *in vivo* in a SGK1-dependent fashion (73). Taken together, these studies show that salt directly alters phenotype and effector functions of CD4^+^ T cells in a microbiota-independent manner.

However, salt has also been reported to have a profound impact on the composition of intestinal microbiota, and only indirectly on the effector functions of intestinal T_H_ cells (91).

Two research groups have independently reported that high salt intake decreases the levels of *Lactobacillus spp*, ultimately favoring inflammation (14, 54). HSD-driven depletion of *Lactobacillus murinus* (*L. murinus*) increased the frequencies of LP T_H_17 cells within small intestine and colon and *L. murinus* supplementation ameliorated EAE by reducing numbers of T_H_17 cells within the spinal cord of mice fed with HSD (14). Importantly, colonization of GF mice either with SFB alone or SFB and *L. murinus* resulted in high or low frequencies of LP T_H_17 cells, suggesting that *L. murinus* presence modulates T_H_17 cells. These findings suggest that dietary habits influence T cell phenotype and their effector functions both in a microbiota-dependent and –independent fashion, determining whether they exhibit protective or pathogenic roles in intestinal immunity.

In addition, HSD has been reported to decrease luminal levels of indole-3-lactic acid (ILA) and butyrate (14, 54). Butyrate promotes the expression of Foxp3, stabilizing the LP T_reg_ phenotype, therefore, its reduction induced by HSD can alter intestinal homeostasis (92).

Taken together, favoring increased lipid, cholesterol and salt consumption leads to alterations of the composition of the intestinal microbiota that in turn affect phenotype and effector function of intestinal CD4^+^ T cells. This can ultimately result in higher susceptibility to both intestinal and extra-intestinal infections and an increased risk of developing chronic autoimmune diseases.

### Fiber, Indoles, and Vitamins

In this part of the review, we describe the effects that WTD-associated low contents of fiber, indoles and vitamins have on adaptive components of the intestinal immune system. Then we propose how supplementation of diets with defined bio-products of bacterial and dietary origin can restore the perturbed intestinal homeostasis. These findings are briefly summarized in Table 2.

**Table 2:** Table showing effects that diet supplementation with defined bio-products can have on CD4^+^ T cell phenotype and on intestinal and extra-intestinal inflammation.

**Dietary supplements**	**Intestinal and extra-intestinal inflammation**	**Effects on CD4^**+**^ T cells**
SCFAs	↓ colitis (93) ↓ small intestinal tumors (*K-ras*^G12Din^) (94)	↑ T_reg_ cells (93, 95) ↑ T_H_17 cells (96)
Indoles/AhR ligands	↑ Resolution of inflammation (35)	↑ T_H_17 cells (97) ↑ T_R_1^exTH17^ cells (35)
Vitamin A	↓ DSS- or TNBS-induced colitis (98, 99)	↓ T_H_17 cells ↑ T_reg_ cells (100, 101) ↑ LP T_reg_ cells (7, 102)
Vitamin D	↓ EAE (103) ↓ clinical symptoms in UC patients^*^ (104)	↓ T_H_17 cells (103)

*AhR, Aryl hydrocarbon Receptor; DSS, Dextran Sulfate Sodium; EAE, Experimental Autoimmune Encephalomyelitis; LP, Lamina Propria; SCFAs, Short-Chain Fatty Acids; TNBS, 2,4,6-Trinitrobenzene Sulfonic Acid; UC, Ulcerative Colitis*.

* *The study showed no amelioration of inflammation*.

**Figure 3:**
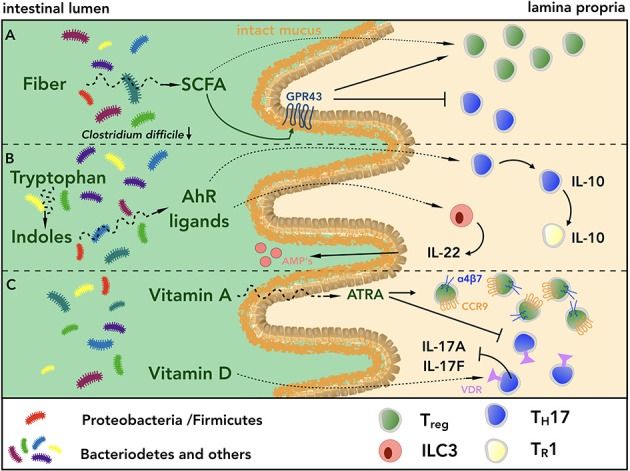
Fiber, indoles and vitamins act in a synergistic fashion promoting intestinal homeostasis. **(A)** Diets rich in fiber promote bacterial species diversity and short chain fatty acid (SCFA)-producing bacteria, which in turn limit pathogen growth, e.g. *Clostridium difficile*, and promote barrier integrity. SCFAs also support intestinal homeostasis upon engagement of GPR43 expressed on intestinal epithelial cells. Furthermore, SCFAs can directly interact with intestinal CD4^+^ T cells, shifting the balance of T_reg_/T_H_17 cells toward T_reg_ cells, thus inducing a regulatory microenvironment. **(B)** Within the lumen of the GI tract, the conversion of indoles to biologically active ligands of AhR contributes to maintain intestinal homeostasis acting on innate and adaptive immune cells. On the one hand, engagement of AhR by its ligands induces secretion of IL-22 from ILC3s, which in turn promotes the production of antimicrobial peptides (AMPs) from Paneth cells. On the other hand, triggering of AhR axis on T_H_17 cells mediates their conversion into IL-10-secreting T_R_1 cells, which are in turn able to terminate immune responses. **(C)** The biologically active form all-*trans* retinoic acid (ATRA) of vitamin A induces the expression of the gut homing receptors CCR9 and α4β7 integrin on T_reg_ cells, contributing to create a regulatory microenvironment within the GI tract, which is further augmented by the reduction of intestinal T_H_17 cells. Similarly, engagement of VDR by active vitamin D metabolites dampens IL-17 secretion by T_H_17 cells.

Individuals with low bacterial species diversity have been shown to exhibit higher body mass index (BMI), serum triglyceride, hemoglobin A1c (HbA1c) and C-reactive protein levels as compared to those with higher diversity, indicating a pivotal role of the intestinal microbiota in maintaining metabolic homeostasis (105, 106). Provision of fiber to the microbial community supports its species diversity (107) and diets low in dietary fiber have been associated with intestinal inflammation (108, 109). The beneficial effects of dietary fiber on mucosal homeostasis are graphically summarized in Figure 3. Deprivation of fiber induces proximity of intestinal bacteria to the epithelium by thinning of mucus layers (110), predisposing to pathogenic infections with *Citrobacter rodentium* (63). Along the same line, mice fed with fiber or inulin alone showed enrichment of SCFA-producing bacteria species, limiting *Clostridium difficile* growth, thus highlighting the therapeutic potential of fiber supplementation (111). In addition, *Kim et al*. showed that mice lacking the G-protein coupled receptor GPR43, one of the main receptors for SCFAs in the intestine, exhibited higher susceptibility to pathogenic infections, DSS-induced colitis and Azoxymethane (AOM)/DSS-induced carcinogenesis, all associated with increased frequencies of colonic LP T_H_17 and decreased frequencies of T_reg_ cells (112, 113). Similarly, in a transfer-colitis model, SCFA supplementation ameliorated intestinal inflammation, increasing T_reg_ cell population in a GPR43-dependent manner (93). Among SCFAs, butyrate has indeed been shown to increase the generation of extra-thymic T_reg_ cells via promoting acetylation of the Foxp3 promoter and the conserved non-coding sequence 1 (CNS1), an enhancer element within Foxp3 locus (95). Butyrate supplementation protected also mice fed with HFD from developing spontaneous small intestinal tumors in the *K-ras*^G12Din^ model (94). Similarly, acetate administration increased the frequencies of IL-17-producing cells during an active immune response to *Citrobacter rodentium*, resulting in augmented bacterial clearance (96).

Taken together, these findings reveal not only the crucial role of SCFAs as mediators of intestinal immunity and mucosal homeostasis through their direct effect on CD4^+^ T cells, but they indirectly point out also the importance of the presence of SCFA-producing bacteria species within the intestinal microbiota.

Besides being characterized by a low content of fiber, WTD is also poor in fruits and vegetables, which have been shown to have a positive impact on human health (114). Green vegetables, especially belonging to the genus *Brassica*, contain indoles that are converted to biologically active ligands of AhR (115). AhR is expressed by various intestinal cell types, including IELs, ILC3s and T_H_17 cells (116, 117). Lack of AhR impairs expression of AMPs, increases gut permeability in DSS-induced colitis and exacerbates immune activation (115). Furthermore, AhR^−/−^ mice exhibit reduction of IL-22-producing ILC3s, leading to higher fitness of SFB that in turn promotes intestinal T_H_17 cells (117, 118). At the same time, the observation that AhR supports T_H_17 cell differentiation through interaction with STAT1 (97), suggests that LP T_H_17 cell development can be mediated by an AhR dependent mechanism. Interestingly, engagement of AhR via 6-Formylindolo[3,2-b]carbazole (FICZ) in *in vitro* differentiated T_H_17 cells induces acquisition of IL-10, favoring their conversion into T_R_1 cells. This indicates that AhR ligands could promote resolution of immune responses (35). It is therefore interesting to speculate that diets rich in green vegetables could limit intestinal inflammation of patients suffering from IBDs via AhR-driven conversion of pro-inflammatory T_H_17 cells into regulatory T_R_1 cells. However, this remains to be proven.

Differentiation and stability of the T_H_17 cell phenotype can also be modulated by vitamins, especially A and D, the contents of which are reduced in WTD. Within the small intestine, dietary vitamin A is converted by CD103^+^ DCs into the biologically active form all-*trans* retinoic acid (ATRA) (119, 120). The detailed roles of ATRA in shaping intestinal immunity have been reviewed elsewhere (121). Administration of ATRA has been shown to ameliorate intestinal inflammation in mice suffering from DSS- or TNBS-induced colitis (98, 99), likely by shifting the balance T_reg_/T_H_17 in favor of T_reg_ cells (100, 101). Addition of ATRA to TGF-β during T_reg_ cell differentiation has also been shown to augment their capacity to migrate to the LP (7) and their *in vivo* suppressive capacities in a murine model of transfer-colitis (100). Of note, mice fed with vitamin A-deficient diet (VAD) showed a substantial decrease in the number of LP CD4^+^ T cells within the small intestine, due to the crucial role of vitamin A in mediating the induction of CCR9 and α4β7 integrin, key gut homing molecules (102). Along the same line, *Tejon et al*. showed that during intestinal inflammation, *in vitro* differentiated T_reg_ cells were able to efficiently convert into T_H_17 cells when transferred into VAD-fed mice, suggesting an anti-inflammatory effect of ATRA (122).

On the one hand, inducing a more regulatory environment within the inflamed mucosa of patients suffering from IBDs via vitamin A supplementation could seem tempting. On the other hand, however, translating its effects in the clinics has been shown to be problematic, and as for now there is no evidence showing beneficial effects of vitamin A supplementation for the health of IBD patients.

Similar to vitamin A, vitamin D content is low in WTD and polymorphisms in the vitamin D receptor (VDR) gene have been associated with higher incidence of IBDs (123). Of note, T_H_17 cells can be sensitive to vitamin D levels given their expression of VDR. In line with this, high intake of vitamin D has been shown to dampen IL-17A and IL-17F secretion of T_H_17 cells, ultimately ameliorating clinical manifestations of EAE (103). Similarly, it has been recently reported that clinical disease activity of patients with active ulcerative colitis (UC) improved after weekly supplementation of cholecalciferol (104). However, no changes in intestinal and systemic inflammation were observed, and other clinical trials involving vitamin D supplementation to patients suffering from IBDs did not show substantial improvement of clinical parameters (124).

Taken together, while these findings question a possible therapeutic role of vitamin supplementation alone in ameliorating intestinal inflammation, they highlight the potential of dietary components in modulating the CD4^+^ T cell phenotype. Among them, SCFAs and AhR ligands could promote intestinal homeostasis and favor mucosal immunity. Translational studies are however required and will eventually shed the light on their efficacy in the clinics.

## Conclusions and Perspectives

Evidences on the impact of biologically active dietary components in modulating mucosal immunity and homeostasis are starting to emerge. Western dietary habits favoring high intake of lipids, cholesterol and salt promote local intestinal and extra-intestinal inflammation shaping phenotype and effector functions of CD4^+^ T cells in a microbiota-dependent or –independent fashion. This can result in altered intestinal immunity, ultimately leading to higher susceptibility to infections caused by intestinal pathogens and increasing the risk for chronic inflammatory autoimmune diseases. The WTD-induced inflammatory state could, however, be potentially reverted by supplementing diets with food rich in fiber and indoles, which represent a promising therapeutic tool to modulate intestinal homeostasis by acting on the T_H_17/T_reg_ cell axis and restoring SCFA-producing bacteria species.

## Author Contributions

FS and NS wrote the manuscript and prepared tables and figures. EV and SH edited the manuscript. NG supervised and edited the manuscript.

### Conflict of Interest Statement

The authors declare that the research was conducted in the absence of any commercial or financial relationships that could be construed as a potential conflict of interest.
